# Pricing strategy research in the dual-channel pharmaceutical supply chain considering service

**DOI:** 10.3389/fpubh.2024.1265171

**Published:** 2024-02-19

**Authors:** Qian Lu, Qing Liu, Yong Wang, Mingke Guan, Zhigang Zhou, Yajun Wu, Jiamin Zhang

**Affiliations:** ^1^Sanya Science and Education Innovation Park of Wuhan University of Technology, Sanya, China; ^2^School of Transportation and Logistics Engineering, Wuhan University of Technology, Wuhan, China; ^3^Department of Production Engineering, KTH Royal Institute of Technology, Stockholm, Sweden; ^4^School of Management, Wuhan University of Science and Technology, Wuhan, China

**Keywords:** pharmaceutical platform supply chain, dual-channel supply chain coordination, complementary products, considering service, game decision-making

## Abstract

In the context of developing a new era, the pharmaceutical supply chain market has gradually transformed from a seller’s market to a buyer’s market. The closer the consumers are, the greater the market pricing power, so the pharmaceutical market power of manufacturers and retailers has also changed. This study considers the effect of service on the pricing strategy of the pharmaceutical platform supply chain. The study aimed to coordinate optimization, and the coordination strategy of the pharmaceutical platform supply chain of complementary products is discussed mainly by researching the price and service factors. Various situations are studied by hypothesis and model solving. This study uses Stackelberg game decision-making. Manufacturers are at the forefront of platform supply chain decisions. The research found that the price was lower under centralized decision-making than under decentralized decision-making. Coordination between price and service levels needs attention in the pharmaceutical platform supply chain of complementary products, and the service level should be controlled within a certain range. Only by improving the service level can enterprises maximize profits, providing a theoretical basis for pharmaceutical supply chain pricing strategy research. Supply chain members must strive to improve service levels to improve medical supply consumers’ (patients) psychological satisfaction level. Service levels do not fully mitigate channel conflict. Therefore, pharmaceutical complements have become a way to alleviate the conflicts in the pharmaceutical platform supply chain.

## 1 Introduction

The outbreak of COVID-19 since January 2020 has disrupted people’s daily lives. The new crown epidemic has profoundly affected people’s travel and economic and social operations. The interconnected technical features of the Internet have improved the cross-regional, non-contact mode of the pharmaceutical supply chain. This made “Internet +” a technological breakthrough for pharmaceutical service systems to respond to the epidemic. The rapidly spreading epidemic has also promoted the development of “Internet +” pharmaceutical industry-related businesses. It has accelerated the internalization of physical hospitals and the rise of platform-based Internet hospitals (1, 2).

Price competition between different entities is involved in the pharmaceutical platform supply chain system. Changes in the price of either party’s product will affect the profits of both parties’ pharmaceutical products. When a party has undergone a detailed market analysis, it will adjust the current price to maximize its profit. While its profit may increase, it may reduce the other party’s profit. As long as the profit of the pharmaceutical platform supply chain is greater than the total profit before adjustment, the supply chain can be optimized through inter-enterprise profit allocation. Coordination has been extensively and deeply studied as a hot issue in optimizing the platform supply chain.

The objective of this study is based on the current development practices of China’s pharmaceutical dual-channel supply chain and studies the impact of service levels on pricing and supply chain coordination in the context of platform supply chains. The pharmaceutical supply chain should control service levels within a certain range and maximize corporate profits by improving consumer satisfaction. Under normal circumstances, an improvement in the service level of either party will lead to an increase in demand for dual channels. However, when consumers are more sensitive to price than service, the increase in demand brought about by improving services will be less than the decrease in demand brought about by increasing price, leading to a continuous decrease in demand. In addition, the sales price of dual-channel complementary products under centralized decision-making will not be higher than that under decentralized decision-making. Finally, this study achieves supply chain coordination through two pricing contracts and Pareto improvement.

The research innovation of this study is mainly reflected in considering the relationship between service level and pricing in the pharmaceutical supply chain; combining the pricing theory in marketing with the conclusions of this study to explain the practical problems that can reflect the market pricing strategy of the pharmaceutical complementary supply chain to a certain extent; and solving the problem that there are few joint studies considering the pricing of complementary products and service cost input in a dual-channel pharmaceutical supply chain system. This study is an effective supplement to the existing research, both in-depth and breadth, and enriches the theoretical and practical research on pharmaceutical dual-channel supply chain pricing.

Cheng et al. (3) point out that Pareto improvement can be achieved by raising prices. Under the background of the platform supply chain, Yang et al. (4) analyzed the price of retail and online channels, order quantity, and delivery time of online channels by considering five decision variables. Barenji et al. (5) considered the effect of simultaneous disruptions of demand and cost on dual-channel supply chain production, pricing, and profit. Zhang et al. (6) studied a dual-channel supply chain system with one manufacturer and two distributors. Iacocca and Mahar (7) studied the effect of pharmaceutical prices, reimbursement contracts, and cost-sharing policies. Ma et al. (8) studied and analyzed the relationship between pharmaceutical device manufacturers’ quality effort level and patient concerns. Chen et al. (9) analyzed the effect of promotional efforts and consumer channel preferences on pharmaceutical supply chain profits. Roy et al. (10) proposed the optimal strategy to maximize customer waiting satisfaction. Martínez et al. (11) analyzed whether enterprises should provide classified services to customers. By introducing the queuing theory, Howard et al. (12) discussed how the government affects the doctor–patient game. Liang et al. (13) modeled and analyzed the queuing network for cooperative referral between upper and lower hospitals within the same pharmaceutical alliance. Liu et al. (14) analyzed China’s pharmaceutical price control policies, motivations, and effects of government pharmaceutical price control. Kouvelis et al. (15) constructed a competitive model among pharmaceutical benefit management companies. Koster et al. (16) studied the effect of pharmaceutical institutions’ public welfare on patients’ self-medication ability. Chen et al. (17) studied the effect of the maximum price policy on different supply chain subjects. Ganuza et al. (18) studied the pharmaceutical pricing problems of two pharmaceutical insurance co-payment modes. Zhang et al. (19) explored the operation strategy selection of e-retailers in the e-commerce mode.

In summary, most current research on the pharmaceutical platform supply chain does not consider the service capability of physical retailers. The queue will be generated when the service capacity does not match the demand. This greatly affects the shopping experience of consumers. At present, the limited service capacity of physical pharmaceutical retailers is a practical problem in developing the pharmaceutical supply chain. This is also a hot issue that needs to be solved. Such problems significantly affect the dual-channel strategy of the OTC pharmaceutical supply chain. By introducing the queuing theory and considering the customer arrival rate, it is of great practical significance to analyze the effect of the service capability of pharmaceutical retailers on the dual channel of the pharmaceutical supply chain. In view of the particularity of pharmaceuticals, with the rapid development of pharmaceutical e-commerce in China, the problem of pharmaceutical platform operation has become more prominent. Scientifically setting dual-channel prices is crucial for pharmaceutical companies to gain competitive advantages in the new era.

## 2 Materials and methods

This study considers the effect of service level on the platform pricing of complementary products in the pharmaceutical supply chain. Pharmaceutical complements are assumed to be strict bidirectional complements. They cost almost the same to produce—a platform supply chain system consisting of manufacturers and distributors. While manufacturers supply raw materials to distributors, they establish direct sales channels in the network. The supply chain sells products supplemented by pharmaceutical raw materials. The equilibrium result expression in the model of this study is too complex to intuitively reflect the optimal pricing strategy under various circumstances, resulting in the inability to reflect the impact of important decision variables on optimal pricing. Therefore, this study uses MATLAB, Excel, and other software to perform numerical calculations and simulations of complex expressions. By analyzing the simulation results of the built model, this study draws corresponding conclusions while verifying the relevant analysis process.

### 2.1 Research methodology

The Stackelberg model is a yield leadership model. There are differences in the order of actions between different subjects. Output is determined according to the following order: The leading manufacturer determines the output. The follower can then observe this output and determine its own output based on the output of the leader. It should be noted that when the leading manufacturer decides its own output, it fully understands how the following manufacturers will act. This means that the leading firm can know the reaction function of the following firm. Therefore, the leading manufacturer will naturally anticipate the impact of its decision on output for the following manufacturers. Taking this influence into account, the output decided by the leading manufacturer will be a profit-maximizing output constrained by the reaction function of the following manufacturer. In the Stackelberg model, the leader’s decision-making no longer requires its own reaction function.

Market competition is not absolutely fair. Companies with rich resources have a greater competitive advantage in the market. In this case, the Stackelberg game decision is considered. The manufacturer is the channel leader, and retailers are not far behind. According to the Stackelberg game decision, the manufacturer seeks to maximize profits. By deriving the retailer’s price response function, the product’s wholesale price and the online channel sales price of the complementary product are further determined. First, the manufacturer determines the wholesale price of the commodity and the online channel sales price of the complementary product according to the optimal response price function of the pharmaceutical retailer. The retailers, as followers, determine their sales prices based on the wholesale price of pharmaceuticals given by manufacturers and the online channel sales prices of complementary pharmaceuticals. The supply chain further maximizes its profits.

### 2.2 Hypothesis

Manufacturers in the pharmaceutical supply chain produce pharmaceuticals A and B, complementary products. The manufacturer provides pharmaceutical B to the retailer. Retailers sell through traditional retail channels. At the same time, the manufacturer sells pharmaceutical A through a variety of means, such as online direct sales. The production cost of pharmaceutical A is $c_{a}$ . The production cost of pharmaceutical B is $c_{b}$ . The wholesale price of pharmaceutical B is w, where $w>c_{b}$ . Their production costs are almost the same. So, let $c_{a}=c_{b}=c$ . The retailer’s retail price for pharmaceutical product B is $p_{b}$ . The manufacturer’s direct selling price for item A is $p_{a}$ . Moreover, $p_{a}$ is greater than the production cost of item A, that is $p_{a}>c$ . Suppose $\alpha_{1}$ is the elasticity coefficient of the effect of the price of the pharmaceutical on demand. $\beta_{1}$ is the elastic coefficient of the cross-price effect of complementary pharmaceuticals. $\gamma_{1}$ is the sensitivity coefficient of the pharmaceutical service. $\gamma_{2}$ is used to represent the cross-sensitivity coefficient between complementary products. The service level provided by the manufacturer is represented by the symbol $s_{m}$ . The level of service the retailer provides is denoted by the symbol $s_{r}$ .

### 2.3 Model solving

Centralized decision-making can avoid the loss of the supply chain in decentralized decision-making. This ensures that supply chain profits are maximized. Both parties take profit maximization of the overall supply chain channel as the ultimate goal of optimization. The two parties work closely together (it can also be seen that the manufacturer and the retailer are two divisions of the same company, and both parties seek to maximize the company’s profits). The profit decision model of the supply chain is:


$$\begin{array}{c} f_{1}=\frac{b\alpha_{1}-a\beta_{1}}{2\alpha_{1}^{2}-2\beta_{1}^{2}},f_{2}=\frac{\gamma_{1}\alpha_{1}+\gamma_{2}\beta_{1}}{2\alpha_{1}^{2}-2\beta_{1}^{2}},\\ f_{3}=\frac{-\gamma_{1}\beta_{1}-\gamma_{2}\alpha_{1}}{2\alpha_{1}^{2}-2\beta_{1}^{2}},f_{4}=\frac{-b\beta_{1}+a\alpha_{1}}{2\alpha_{1}^{2}-2\beta_{1}^{2.}} \end{array}$$

Substituting $p_{a}^{*}$ and $p_{b}^{*}$ into the centralized decision-making model of the platform supply chain can get the optimal profit $\pi_{sc}$ and the demand $D_{m}$ and $D_{r}$ in this case. However, its expression is more complicated and is not given here. When $\alpha_{1}>\beta_{1}>0$ , according to the definition of equation (5), the supply chain profit $\pi_{sc}$ in the case of concentration is a joint concave function about $p_{a}$ and $p_{b}$ . This formula has a maximum profit of about $p_{a}$ and $p_{b}$ , and the maximum point is ( $p_{a}^{*},p_{b}^{*}$ ).

The retailer’s optimal selling price in traditional channels can be obtained.


$$p_{b}^{**}=\left(f_{2}+\frac{\gamma_{1}}{4\alpha_{1}}\right)s_{r}+\left(f_{3}+\frac{\gamma_{2}}{4\alpha_{1}}\right)s_{m}+\frac{\alpha_{1}-\beta_{1}}{4\alpha_{1}}\cdot c+f_{1}+\frac{b}{4\alpha_{1}}$$

There is an equivalence relationship between the retail price of the centralized decision and the manufacturer’s wholesale price of the decentralized decision. There is an equivalence relationship between the manufacturer’s online price for centralized decision-making and the manufacturer’s online direct selling price for decentralized decision-making. In this supply chain system, the retail price of retailers will be lower in the centralized case than in the decentralized case. In a centralized situation, consumers can obtain greater price concessions. In the Stackelberg game process dominated by the manufacturer, the manufacturer’s profit reaches its maximum value at point ( $p_{a}^{**},w^{**}$ ). The retailer’s profit reaches its maximum value at $p_{b}^{**}$ .

Manufacturers’ wholesale prices are inversely related to the level of service they provide. In the retail channel, the wholesale price of complementary pharmaceuticals will gradually decrease with the manufacturer’s service level improvement. At the same time, there is a positive correlation between the manufacturer’s wholesale price and the retailer’s service level. That is, with the improvement of the retailer’s service level, the wholesale price of ancillary products provided by the manufacturer gradually increases. Whether centralized or decentralized, the manufacturer’s direct selling price is positively related to its service level. That is, by improving the level of service, the price of its products will also increase accordingly. At the same time, a retailer’s retail price is positively correlated with its service level.

For centralized decision-making, channel demand is positively related to service level. At the same time, there is a positive correlation between the channel’s demand and the complementary pharmaceutical service level in the competitive channel. The higher the service levels of complementary pharmaceuticals in the competitive channel, the greater the product demand for this channel. Under decentralized decision-making, the relationship between the channel demand of manufacturers (retailers) and the service level of complementary products of pharmaceutical retailers (manufacturers) depends on the relationship between $\gamma_{1}$ and $\gamma_{2}$ . When $\gamma_{2}<\gamma_{1}<6\gamma_{2}$ , the demand of traditional channel retailers is positively correlated with the service level of supporting products of direct sales channel manufacturers. The direct sales channel manufacturers’ demand positively correlates with the retailer’s supporting product service level. When $\gamma_{1}>6\gamma_{2}$ , the demand of manufacturers in direct sales channels is negatively correlated with the level of complementary products and services provided by pharmaceutical manufacturers in direct sales channels.

## 3 Results

In this study, the study of a pharmaceutical dual-channel supply chain system involves different price competition, and the change in price on either side will have an impact on the profits of both sides. After a detailed market analysis, one party will adjust the current price to maximize its own profit, which may reduce the other party’s profit while increasing its own profit. As long as the profit of the dual-channel pharmaceutical supply chain is greater than the profit sum before adjustment, the supply chain can be optimized through profit allocation.

### 3.1 Numerical simulation

First, the relationship between pricing, wholesale price, demand, and service level of complementary products in the supply chain of pharmaceutical platforms is analyzed. Next, the relationship between service level and platform membership is further verified and analyzed by numerical simulation.

**Figure 1:**
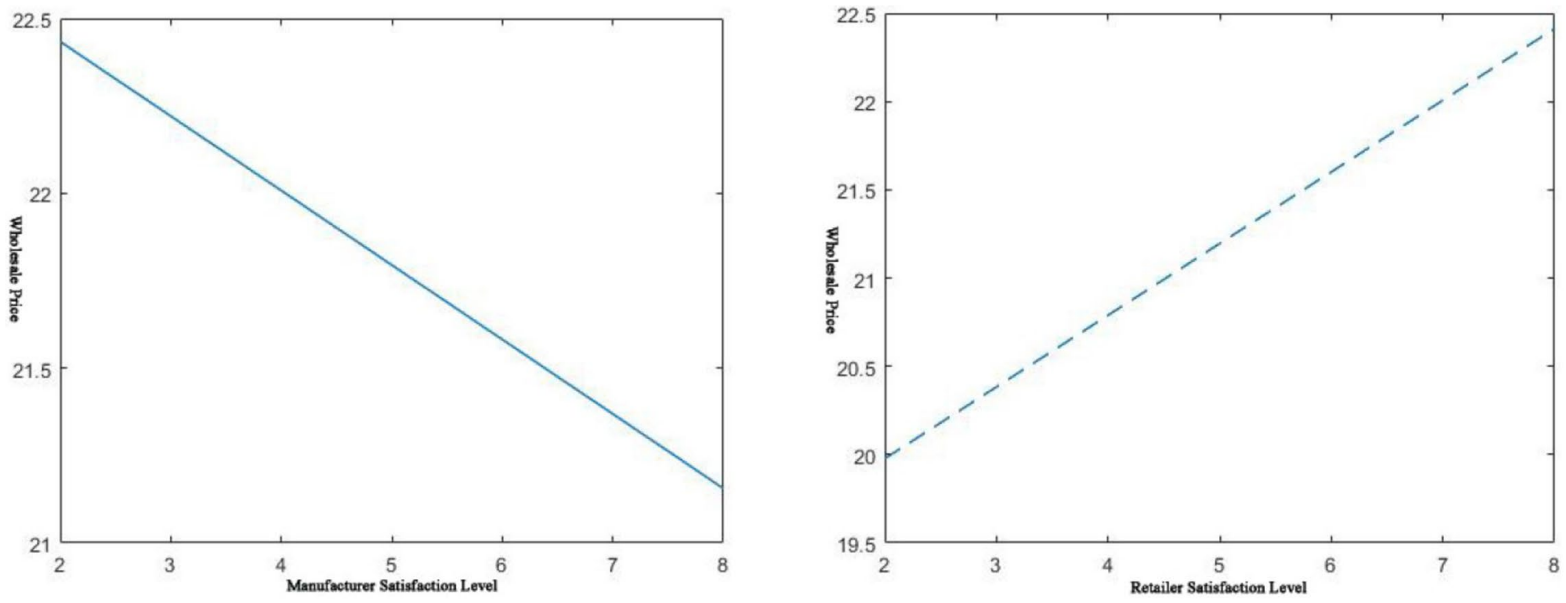
𝑠_𝑚_, 𝑠_𝑟_ effect on wholesale prices.

**Figure 2:**
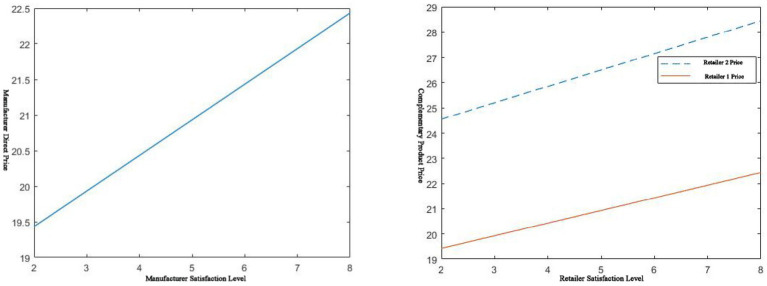
𝑠_𝑚_, 𝑠_𝑟_ effect on direct selling prices.

In the platform supply chain of complementary pharmaceuticals, assumption $a=400,b=400,\alpha_{1}=10,\beta_{1}=5,\gamma_{1}=6,\gamma_{2}=3,c=15$ , When $s_{m}=5$ , $s_{r}\in \left[2,8\right],s_{r}=5,s_{m}\in \left[2,8\right]$ , for this set of assignments, satisfy $\alpha_{1}>\beta_{1}>0,\gamma_{1}>\gamma_{2}>0$ . Then there is the effect of service level on the manufacturer’s wholesale price, as shown below in Figures 1, 2.

As shown in the left of Figure 1, when the manufacturer’s service level increases, the wholesale price of the complementary products it provides will decrease. This shows that when a manufacturer has a large inventory of a certain commodity, it can stimulate the demand for traditional retail channels by improving the service level of its complementary pharmaceuticals. Lowering the wholesale price of the item causes retailers to stock up in bulk to clear inventory quickly. It can be seen from the right of Figure 1 that the higher the dealer’s service level, the higher the manufacturer’s wholesale price. As retailers ramp up their service levels, demand will increase rapidly. Due to the limitation of production capacity, manufacturers must increase the cost of their workforce and material resources. This would lead to higher costs for manufacturers and higher wholesale prices for the commodity.

As shown on the left of Figure 2, the higher the manufacturer’s service level, the higher the price of its goods. The higher the input cost, the higher the price will inevitably be. However, at the same time, this situation improves the patient’s consumption and purchase experience, allowing the patient to obtain greater utility. In this situation, the manufacturer employs high-service-level, cost-effective strategies for rapid skimming. The manufacturer dominates the market and returns on investment. Conversely, its selling price is also low when the service level is low. Currently, the pharmaceutical manufacturer adopts a slow penetration strategy for pricing. The choice of the above two pricing methods depends on the item’s life cycle. During the introduction period of the product, a rapid skimming strategy is employed. In the period of commodity decline, the penetration strategy was adopted to gradually eliminate skinny dog products and pay more attention to star products. As can be seen from the right of Figure 2, pharmaceutical retailers will also adopt a rapid skimming strategy or a slow penetration strategy according to the situation. Similarly, the choice of a retailer’s pricing strategy will still be determined by the product’s life cycle. At the same time, according to Figure 2, it can be seen that the commodity pricing in centralized decision-making is not greater than that in decentralized decision-making.

**Figure 3:**
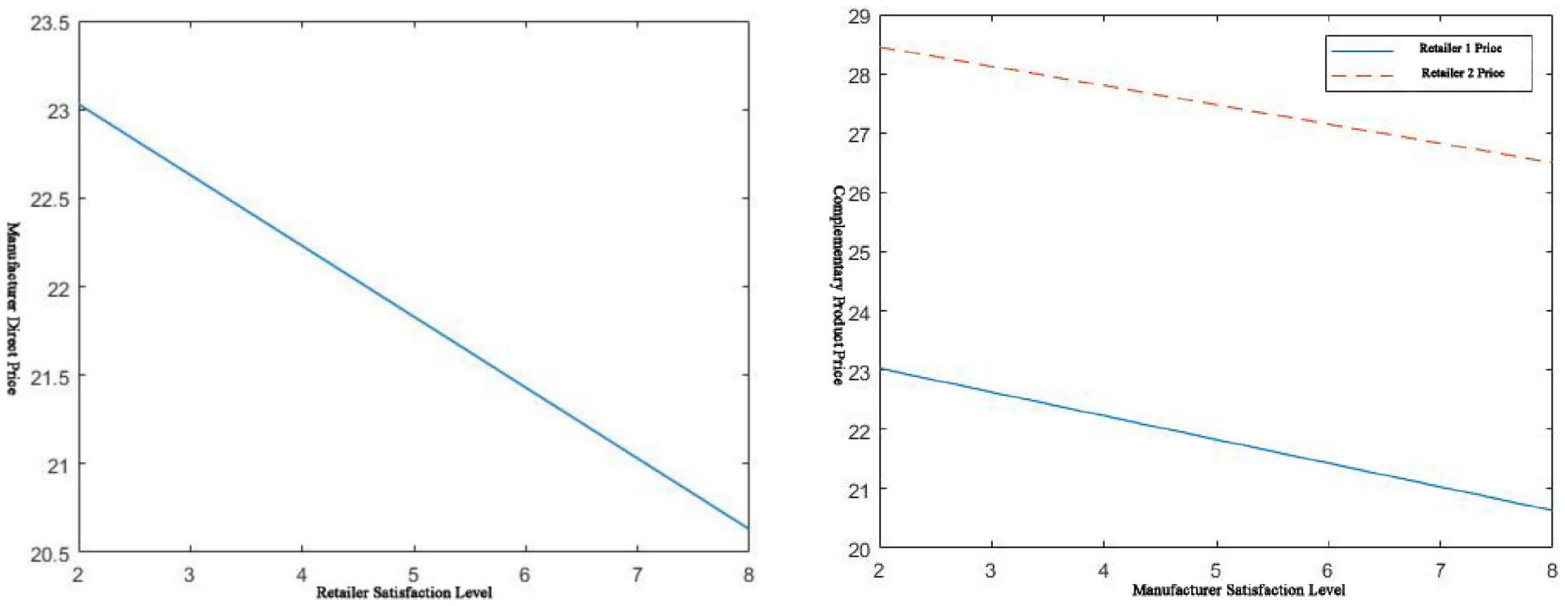
𝑠_𝑚_, 𝑠_𝑟_ effect on direct selling prices.

The effect of service level on the price of the other party is shown in Figure 3. Whether it is a manufacturer or a retailer, the service level of either party is improved.

The other party will take price reduction measures to quickly respond to market demand to achieve small profits but quick turnover. It can also be understood that when the manufacturer adopts a rapid skimming strategy, the retailer will adopt a mixed rapid penetration strategy because of the manufacturer’s high service level and the retailer’s low price. Growing commodities are more suitable for this strategy. When the manufacturer adopts a slow penetration strategy, the retailer adopts a slow skimming strategy, mixed with the manufacturer’s low service level and high price. This strategy applies to commodities in the mature stage. When the retailer adopts a rapid skimming strategy, the manufacturer adopts a hybrid rapid penetration strategy based on the retailer’s high service level and the manufacturer’s low price. From Figure 3, it can also be concluded that the price in the case of centralized decision-making by both parties in the channel is not higher than the price of decentralized decision-making. This also reflects the benefits of centralized decision-making.

**Figure 4:**
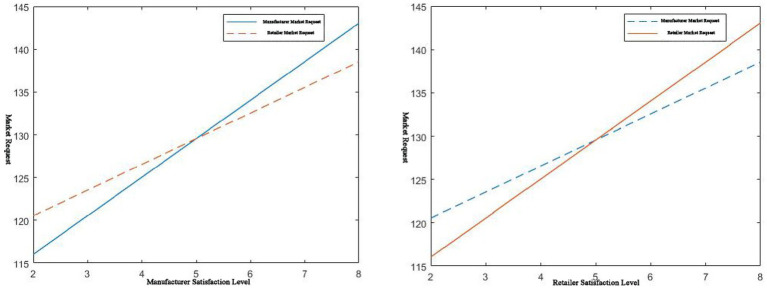
𝑠_𝑚_, 𝑠_𝑟_ effect on market demand.

As can be seen from Figure 4, in centralized decision-making, no matter which party’s service level is improved, it will increase platform supply chain market demand. This is due to the particularity of complementary goods. Complementary goods are products whose two are combined to obtain their utility. The improvement of the service level of a commodity will inevitably bring about an increase in its sales volume, as well as an increase in the sales volume of its complementary products. Positive spillover effects between complements lead to this outcome. When service improves, platform demand increases; while service decreases, platform demand decreases. For example, pharmaceutical supplies and remote-control software are complementary products. Pharmaceutical manufacturers launch remote control software services. If hospitals or pharmaceutical retailers support this service, the sales of remote terminal software and pharmaceutical supplies will increase accordingly. At the same time, it can be seen from Figure 4 that its service level has a greater effect on its market demand. This is also in line with market laws. It appears that the market demand for it is smaller than the market demand for the other party because it is the demand when the service level of the other party is fixed. For example, on the left of Figure 4, $s_{r}=5$ is a fixed service level, and $s_{m}$ varies in the range of (2, 8). When $s_{m}>5$ , the manufacturer’s market demand is larger and faster.

**Figure 5:**
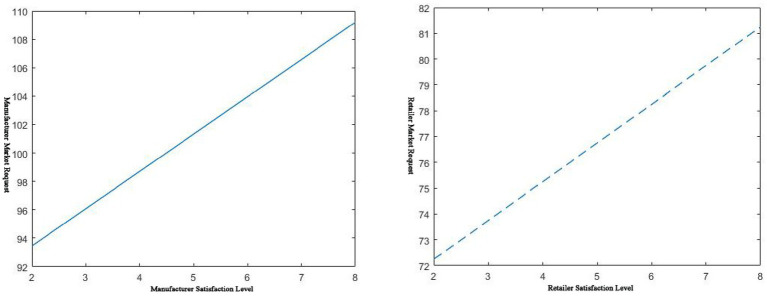
𝑠_𝑚_, 𝑠_𝑟_ effect on own market demand.

According to Figure 5, in the case of decentralized decision-making, there is also a positive correlation between service level and demand. In other words, the relationship between them can be expressed as the higher the service level, the greater the market demand itself.

**Figure 6:**
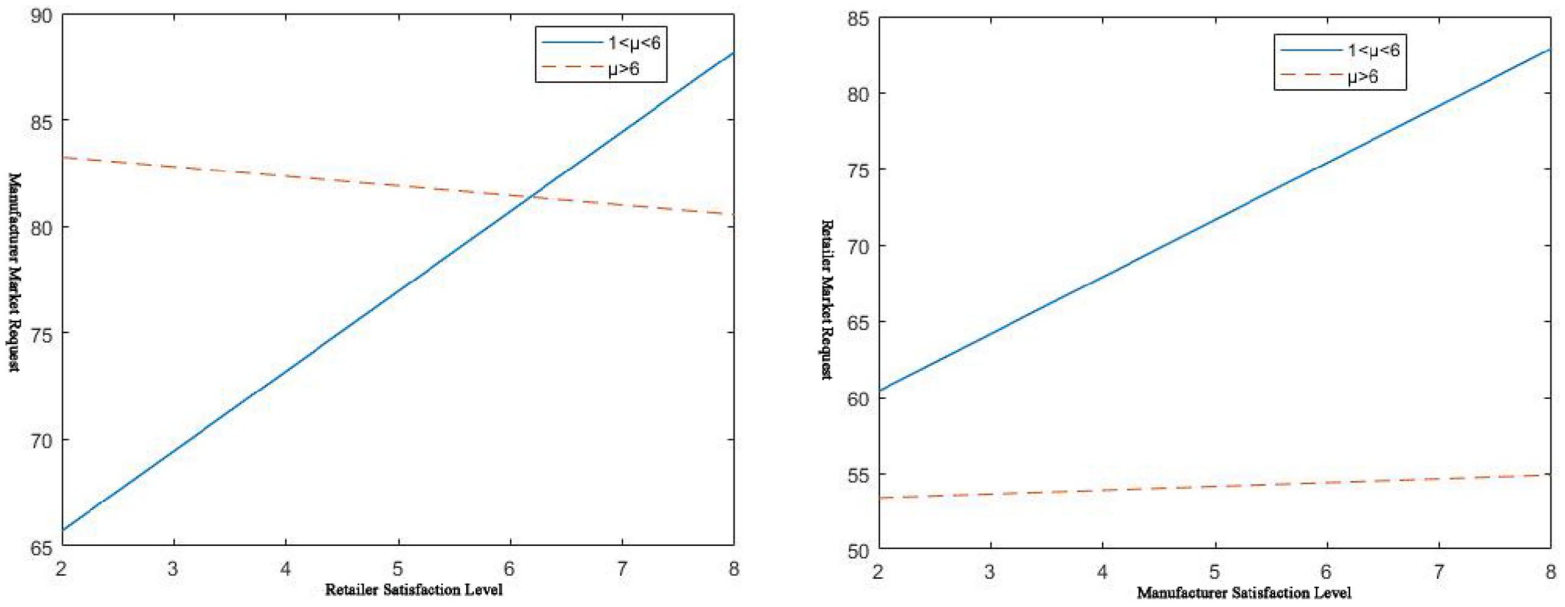
𝑠_𝑚_, 𝑠_𝑟_ effect on manufacturer market demand/retailer market demand.

In the case of decentralized decision-making, the effect of service level on the market demand of the other party can be divided into two situations. When $\mu =\frac{\gamma_{1}}{\gamma_{2}},\gamma_{2}<\gamma_{1}<6\gamma_{2}$ , which is 1<μ<6 . When $\gamma_{1}>6\gamma_{2}$ , which is μ>6 . The previous data satisfy 1<μ<6 . So just put $\gamma_{1}$ and $\gamma_{1}$ change accordingly. Assumed $\gamma_{1}=6,\gamma_{2}=0.2$ , then satisfied μ>6 , The corresponding change trend is as follows in Figure 6.

It can also be seen from Figure 6 that when 1 < μ < 6, the market demand of both channels shows a relatively obvious positive correlation with the service level of the other party. When μ > 6, the manufacturer’s market demand negatively correlates with the retailer’s service level. However, the retailer’s market demand increases with the manufacturer’s service level improvement. The channel’s demand will increase with the improvement of its service level. Under the premise of satisfying 1 < μ < 6, both parties of the channel will ask the other party to improve their service level while improving their service level. Under the premise of satisfying μ > 6, manufacturers tend to require retailers to have lower service levels. Furthermore, retailers tend to demand a higher level of service from manufacturers. The improvement in service level will bring about a rapid increase in cost. Therefore, the decision on service fees becomes the next issue to be considered. Both pricing and service costs are considered in the pharmaceutical platform supply chain of complementary products.

### 3.2 Research on coordination mechanism

References show that the sum of the profits of both sides of the pharmaceutical supply chain channel is always smaller than the total profit of the supply chain when the decision is centralized. In the absence of a contract as a coordination mechanism, both sides of the channel, starting from the perspective of their profit maximization and the conflicting goals of the two parties, will lead to an imbalance in the supply chain. Therefore, in order to improve the performance of the platform supply chain and increase the profits of both channels, a two-part pricing coordination mechanism is proposed.

First of all, as the leading pharmaceutical supply chain channel enterprise, the manufacturer first wholesales complementary products to pharmaceutical retailers at a lower price. Incentivize retailers while reducing inventory expenses. The retailer will compensate the manufacturer with a fixed fee to protect the manufacturer’s profit. Under this contract mechanism, it is assumed that the wholesale price of complementary pharmaceuticals is $w^{c}$ . The manufacturer’s direct selling price is $p_{a}^{c}$ . The retailer’s retail price is $p_{b}^{c}$ . The retailer’s compensation fee to the manufacturer is f , without loss of generality, $p_{b}^{c}>w^{c}$ .

Both sides of the channel continue to play the Stackelberg game, dominated by the manufacturer. The decision model can be expressed as Equation 13.


$$\max\;\pi_{m}^{c}\left(p_{a}^{c},w^{c}\right)=\left(a-\alpha_{1}p_{a}^{c}-\beta_{1}p_{b}^{c}+\gamma_{1}s_{m}+\gamma_{2}s_{r}\right)\left(p_{a}^{c}-c\right)$$

(13) $$+\left(w^{c}-c\right)\left(b-\alpha_{1}p_{b}^{c}-\beta_{1}p_{a}^{c}+\gamma_{1}s_{r}+\gamma_{2}s_{m}\right)-\frac{1}{2}\eta s_{m}^{2}+f$$

(14) $$s.t.\max\;\pi_{r}^{c}\left(p_{b}^{c}\right)=\left(p_{b}^{c}-w^{c}\right)\left(\begin{array}{l} \;b-\alpha_{1}p_{b}^{c}-\beta_{1}p_{a}^{c}\\ +\gamma_{1}s_{r}+\gamma_{2}s_{m} \end{array}\right)-\frac{1}{2}\eta s_{r}^{2}$$

Let equation (14) be equal to zero, and the reaction function of $p_{b}^{c}$ about $p_{a}^{c},w^{c}$ can be obtained.


(15) $$p_{b}^{c}=\frac{1}{2\alpha_{1}}\left(\alpha_{1}w^{c}-\beta_{1}p_{a}^{c}+b+\gamma_{1}s_{r}+\gamma_{2}s_{m}\right)$$

Assuming that the contract can realize the coordination of the platform supply chain of complementary pharmaceuticals, there must be: $p_{a}^{c}=p_{a}^{*},p_{b}^{c}=p_{b}^{*}$ . It can also be seen that $p_{a}^{c}=p_{a}^{*},p_{b}^{*}=w^{**}$ , Combine the above conditions $p_{b}^{c}>w^{c}$ , So $p_{b}^{c}=p_{b}^{*}=w^{**}>w^{c}$ , the wholesale price after coordination is less than the wholesale price when the decision is decentralized.

Substitute $p_{a}^{c}=p_{a}^{*},p_{b}^{c}=p_{b}^{*}$ , into Equation (15), the coordinated wholesale price can be obtained as shown in Equation 16:


(16) $$w^{c}=2p_{b}^{*}-\frac{b-\beta_{1}p_{a}^{*}+\gamma_{1}s_{r}+\gamma_{2}s_{m}}{\alpha_{1}}$$

The contract realizes the coordination of the platform supply chain, but a good contract can promote the improvement of the profits of both channels. Therefore, for both parties to be willing to accept the contract, the profit after coordination must be greater than the profit before coordination, that is, $\pi_{m}^{c}>\pi_{m}^{**}$ , $\pi_{r}^{c}>\pi_{r}^{**}$ . In order to illustrate the size of the compensation fee f, the numerical value is divided into two cases.

1. When 1< *μ* < 6, let a = 400, b = 400, α_1_ = 10, β_1_ = 5, γ_1_ = 6, γ_2_ = 3, c = 15, then the size of the corresponding compensation fee f is shown in Table 1:
2. When  *μ* > 6, let a = 400, b = 400, α_1_ = 10, β_1_ = 5, γ_1_ = 6, γ_2_ = 0.2, the size of the corresponding compensation fee is shown in Table 2:

**Table 1:** 1< *μ* < 6, Service Difference Compensation Fee *f*.

Model type	p_a_	p_b_	w	π_m_	π_r_	π_sc_
Centralized decision-making	21.733	20.833	N/A	N/A	N/A	1510.733
Decentralized decision-making	21.733	26.183	20.833	1023.333	361.675	1385.008
After the coordination	21.733	20.833	8.633	27.133+ *f*	1483.600- *f*	1510.733
$s_{m}=5>4=s_{r}$			value of *f*	996.2 < *f* < 1121.925		

Model type	p_a_	p_b_	w	π_m_	π_r_	π_sc_
Centralized decision-making	21.333	21.333	N/A	N/A	N/A	1568.333
Decentralized decision-making	21.333	26.833	21.333	1053.333	377.500	1430.833
After the coordination	21.333	21.333	8.833	13.333 + *f*	1555.000-*f*	1568.333
$s_{m}=5=5=s_{r}$			value of *f*	1040 < *f* < 1175.5		

Model type	p_a_	p_b_	w	π_m_	π_r_	π_sc_
Centralized decision-making	20.933	21.833	N/A	N/A	N/A	1624.733
Decentralized decision-making	20.933	27.483	21.833	1081.833	393.175	1475.008
After the coordination	20.933	21.833	9.033	−2.867 + *f*	1627.600 + *f*	1624.733
$s_{m}=5<6=s_{r}$			value of *f*	1087.4 < *f* < 1234.425		

**Table 2:** *μ* < 6, Service Difference Compensation Fee *f*.

Model type	p_a_	p_b_	w	π_m_	π_r_	π_sc_
Centralized decision-making	22.013	21.393	N/A	N/A	N/A	1360.709
Decentralized decision-making	22.013	26.393	21.393	870.509	250.200	1120.709
After the coordination	22.013	21.393	11.293	345.409+ *f*	1015.300- *f*	1360.709
$s_{m}=5>4=s_{r}$			value of *f*	525.1 < *f* < 765.1		

Model type	p_a_	p_b_	w	π_m_	π_r_	π_sc_
Centralized decision-making	21.800	21.800	N/A	N/A	N/A	1399.400
Decentralized decision-making	21.800	26.950	21.800	881.600	262.875	1144.475
After the coordination	21.800	21.800	11.400	325.300+ *f*	1074.100- *f*	1399.400
$s_{m}=5=5=s_{r}$			value of *f*	556.3 < *f* < 811.425		

Model type	p_a_	p_b_	w	π_m_	π_r_	π_sc_
Centralized decision-making	21.587	22.207	N/A	N/A	N/A	1439.803
Decentralized decision-making	21.587	27.507	22.207	894.103	275.400	1169.503
After the coordination	21.587	22.207	11.507	305.703+ *f*	1134.100- *f*	1439.803
$s_{m}=5<6=s_{r}$			value of *f*	588.4 < *f* < 858.7		

From the analysis of Tables 1, 2, it can be concluded that when 1 < *μ* < 6 and  *μ* < 6, Harmonization and Pareto improvement can be achieved through both pricing contracts. However, there are differences in the effects of coordination. When 1 < *μ* < 6, manufacturers’ profits have fallen sharply. Moreover, when the manufacturer’s service level is low ( $s_{m}=5<6=s_{r}$ ), in the case that the retailer does not compensate for the fixed cost, to ensure the Pareto improvement of the manufacturer’s profit, it is necessary to realize that the manufacturer has absolute dominance over the retailer. The retailer is compelled to comply with the contract through this right. Otherwise, it is difficult to guarantee the profit of the manufacturer.

When *μ* > 6, on the one hand, the profit reduction of the manufacturer (manufacturer) is smaller. On the other hand, the profit growth of retailers is also relatively small. In this case, the two-part pricing contract is relatively firm, and the manufacturer is more willing to accept it. At the same time, by comparing Tables 1, 2, under the premise that other conditions remain unchanged, the total profit of the supply chain when 1 < *μ* < 6 is higher. Manufacturers that are market leaders are also higher.

**Figure 7:**
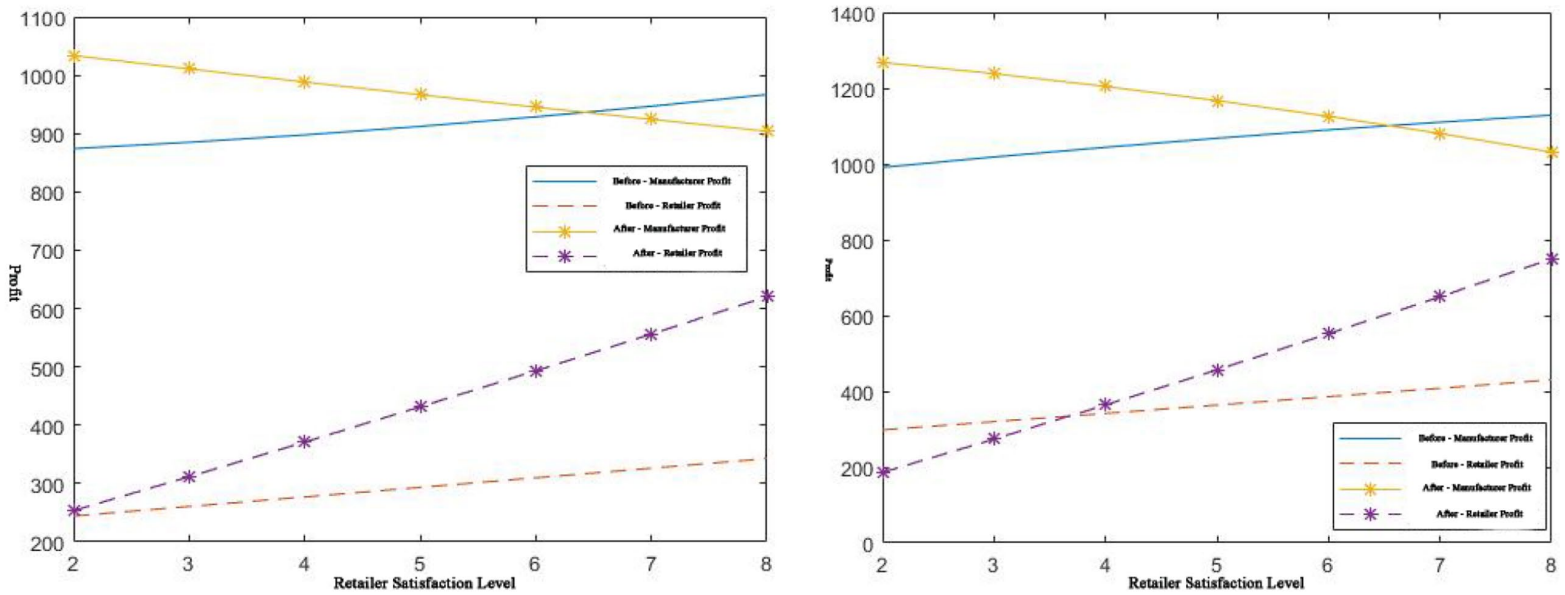
*μ* > 6 / 1 < *μ* < 6, effect on profit before and after coordination.

Therefore, manufacturer decisions depend on absolute market power and risk attitudes. The following mainly verify Tables 1, 2. Take *η* = 0.6, when μ>6,letf=700 , when 1<μ<6 , let f=1100 , then the corresponding profit coordination comparison is shown in Figure 7:

It can be seen from Figure 7 that when μ>6 , $s_{r}$ is at (2, 6) when the manufacturer’s service level is constant ( $s_{m}=5$ ). Through the two pricing strategies, the coordination of platform supply chains of complementary products can be achieved, and Pareto improvement can be achieved. In this case, the compensation fee is relatively low.

When 1<μ<6 , in the case of a certain manufacturer’s service level $(s_{m}=5$ ), $s_{r}$ is at (4, 6), and through two pricing strategies, Pareto improvement can be achieved through further supply chain coordination. In this case, the compensation cost is relatively high, and a Pareto improvement is achieved. The improved retailer’s service level has more stringent input conditions, which verifies Tables 1, 2.

## 4 Discussion

Based on the above simulation process and the relationship between price and service level, pharmaceutical manufacturers, depending on their life cycle, tend to adopt a high-service-level and high-sale-price quick skimming strategy for medicine in the introduction period. For medicine in the declining stage, pharmaceutical manufacturers tend to choose a slow penetration strategy of low service level and low sale price. Correspondingly, when pharmaceutical manufacturers adopt a rapid skimming strategy, pharmaceutical retailers will adopt a high-service-level and low-sale-price hybrid rapid penetration strategy, which is suitable for medicine in the growth stage.

When pharmaceutical manufacturers adopt a slow penetration strategy, pharmaceutical retailers adopt a low-service and high-sale-price hybrid slow skimming strategy, which is suitable for medicine in its mature stages. Under normal circumstances, an improvement in the service level of either party will lead to an increase in demand for dual channels. However, when patients are more sensitive to price than service, the increase in demand brought about by improving services will be less than the decrease in demand brought about by increasing price, which will lead to a continuous decrease in demand. In addition, the sales price of dual-channel pharmaceutical complementary products under centralized decision-making will not be higher than the sales price under decentralized decision-making. Finally, supply chain coordination is achieved through two pricing contracts, and Pareto improvement is achieved.

This study considers the relationship between price and service level in the pharmaceutical dual-channel supply chain of complementary products under an ideal situation. This study does not consider the impact of inventory during production operations and stock-outs on prices. Therefore, the limitations of the study are as follows:

1. This study does not consider inventory as an important influencing factor in the pharmaceutical dual-channel supply chain of complementary products. A stochastic demand function is not used in the selection of the demand function to better reflect the relationship between service level and complementary product inventory, service level and complementary product pricing, and profit. In the future, research on this topic will require the design of corresponding contracts to achieve performance improvements in complementary dual-channel products.
2. The study does not combine the latest methods and theories in the fields of machine learning and data science to verify the effectiveness and stability of the model. The study does not conduct an in-depth evaluation of its accuracy and consistency in practical applications through further application of large-scale group experimental data.

## 5 Conclusion

This study mainly studied the effect of service level between complementary products on pricing and the coordination of the pharmaceutical platform supply chain. It was found that the price is lower when centralized decision-making is compared with decentralized decision-making. In decentralized decision-making, a manufacturer’s wholesale price is inversely proportional to its service level and the service level of a pharmaceutical retailer selling complementary products. Improved service levels will increase demand across channels. This situation correspondingly increases the commodity’s selling price and reduces the selling price of its complementary pharmaceuticals.

Through the two-part pricing strategy, coordination in the dual-channel supply chain of pharmaceutical complementary products can be achieved, allowing both channels to achieve Pareto improvement. However, the service levels of manufacturers and retailers are only used as influencing factors, not as decision variables. Service level directly determines the level of service costs. In the dual-channel supply chain of pharmaceutical complementary products, attention should be paid to the coordination between prices and service levels between complementary products, and the service level should be controlled within a certain range. Only by improving the service level can the company’s profits be maximized.

## Data availability statement

The datasets presented in this study can be found in online repositories. The names of the repository/repositories and accession number(s) can be found in the article/supplementary material.

## Author contributions

QiaL: Software, Writing – original draft. QinL: Investigation, Writing – review & editing. YoW: Validation, Writing – original draft. MG: Visualization, Writing – review & editing. ZZ: Data curation, Writing – review & editing. YaW: Resources, Writing – review & editing. JZ: Project administration, Writing – review & editing.

## References

[1] Filip R Gheorghita Puscaselu R Anchidin-Norocel L Dimian M Savage WK. Global challenges to public health care systems during the COVID-19 pandemic: a review of pandemic measures and problems. J Personal Med. (2022) 12:1295. doi: 10.3390/jpm12081295, PMID: 36013244 PMC9409667

[2] Feng W Zhang LN Li JY Wei T Peng TT Zhang DX. Analysis of special ehealth service for corona virus disease 2019 (COVID-19) pneumonia. Beijing da xue xue bao Yi xue ban. (2020) 52:302–7. doi: 10.19723/j.issn.1671-167X.2020.02.018 PMID: 32306015 PMC7433451

[3] Cheng X Gou Q Yue J Zhang Y. Equilibrium decisions for an innovation crowdsourcing platform. Transport Res Part E. (2019) 125:241–60. doi: 10.1016/j.tre.2019.03.006

[4] Yang H Peng J. Coordinating a fresh-product supply chain with demand information updating: Hema fresh O2O platform. RAIRO-Operations Res. (2021) 55:285–318. doi: 10.1051/ro/2021024

[5] Barenji AV Wang WM Li Z Guerra-Zubiaga DA. Intelligent E-commerce logistics platform using hybrid agent based approach. Transport Res Part E. (2019) 126:15–31. doi: 10.1016/j.tre.2019.04.002

[6] Xuelong Z Junjin W Jiaguo L. Research on coordination decision-making model of supply chain influenced by Price and service double factors. Operations Research and Management. (2018) 27:57–65. doi: 10.12005/orms.2018.0206

[7] Iacocca KM Mahar S. Cooperative partnerships and pricing in the pharmaceutical supply chain. Int J Prod Res. (2019) 57:1724–40. doi: 10.1080/00207543.2018.1504249

[8] Ma P Gong Y Jin M. Quality efforts in medical supply chains considering patient benefits. Eur J Oper Res. (2019) 279:795–807. doi: 10.1016/j.ejor.2019.06.030

[9] Xiaochun C Zhang Wensong Weijun G. Research on coordination of pharmaceutical supply chain considering promotion behavior and consumer preference. Ind Eng Manag. (2019) 24:24–42.

[10] Roy D Bandyopadhyay A Banerjee P. A nested semi-open queuing network model for analyzing dine-in restaurant performance. Comput Oper Res. (2016) 65:29–41. doi: 10.1016/j.cor.2015.06.006

[11] Martínez-Espiñeira R García-Valiñas MA González-Gómez F. Is the pricing of urban water services justifiably perceived as unequal among Spanish cities? Int J Water Res Dev. (2012) 28:107–21. doi: 10.1080/07900627.2012.642231

[12] Howard DH Bach PB Berndt ER Conti RM. Pricing in the market for anticancer drugs[J]. J Econ Perspect. (2015) 29:139–62. doi: 10.1257/jep.29.1.139 28441702

[13] Liang H Tsui BY Ni H Valentim CCS Baxter SL Liu G. Evaluation and accurate diagnoses of pediatric diseases using artificial intelligence[J]. Nat Med. (2019) 25:433–8. doi: 10.1038/s41591-018-0335-9 30742121

[14] Liu GG Vortherms SA Hong X. China's health reform update. Annu Rev Public Health. (2017) 38:431–48. doi: 10.1146/annurev-publhealth-031816-044247 28125384

[15] Kouvelis P Xiao Y Yang N. PBM competition in pharmaceutical supply chain: formulary design and pharmaceutical pricing. Manuf Serv Oper Manag. (2015) 17:511–26. doi: 10.1287/msom.2015.0542

[16] Koster ES Philbert D Bouvy ML. Impact of the COVID-19 epidemic on the provision of pharmaceutical care in community pharmacies. Res Soc Adm Pharm. (2021) 17:2002–4. doi: 10.1016/j.sapharm.2020.07.001, PMID: 33317768 PMC7330552

[17] Chen X Yang H Wang X. Effects of price cap regulation on the pharmaceutical supply chain. J Bus Res. (2019) 97:281–90. doi: 10.1016/j.jbusres.2018.01.030

[18] Ganuza JJ Llobet G Domínguez B. R&D in the pharmaceutical industry: a world of small innovations. Manag Sci. (2009) 55:539–51. doi: 10.1287/mnsc.1080.0959

[19] Zhang H He QM Zhao X. Balancing herding and congestion in service systems: a queueing perspective. INFOR. (2020) 58:511–36. doi: 10.1080/03155986.2020.1734902

